# Synthesis of Perovskite by Solid-Phase Method with Metatitanic Acid and Calcium Carbonate and Its Pigment Properties Investigation

**DOI:** 10.3390/ma13071508

**Published:** 2020-03-26

**Authors:** Han Zhang, Sijia Sun, Wei Liu, Hao Ding, Jianmeng Zhang

**Affiliations:** Beijing Key Laboratory of Materials Utilization of Nonmetallic Minerals and Solid Wastes, National Laboratory of Mineral Materials, School of Materials Science and Technology, China University of Geosciences, Xueyuan Road, Haidian District, Beijing 100083, China; zhanghan0050@163.com (H.Z.); ssjcugb@163.com (S.S.); 2103150001@cugb.edu.cn (W.L.); 2103180006@cugb.edu.cn (J.Z.)

**Keywords:** metatitanic acid, calcium carbonate, perovskite, pigment, calcination

## Abstract

Synthetic perovskite powder (SPP) was synthesized by the solid-phase method using metatitanic acid (TiO_2_·nH_2_O) and calcium carbonate (CaCO_3_) as raw materials, and its structure, morphology, pigment properties and application in architectural coatings were studied. When TiO_2_·nH_2_O and CaCO_3_ were mixed and ground at a molar ratio of TiO_2_:CaO = 1:1, and then calcined at 900–1100 °C, SPP with a single perovskite phase was obtained. The characterization results displayed that the unit particle size of SPP was 50–150 nm, the aggregate size was 1–2 μm, and its particles were well dispersed. The SPP also had a whiteness of 90.5%, and an oil absorption of 35.03 g/100 g. The hiding power of SPP was 24.02 g/m^2^, which was 81.6% of pure TiO_2_ hiding power (19.60 g/m^2^). When adding SPP to prepare a building exterior wall coating, the contrast ratio of the coating film was 0.92, which met the requirements of the Chinese national standard GB/T 9755-2014 and was equivalent to adding rutile titanium dioxide. Thus, perovskite synthesized from TiO_2_·nH_2_O and CaCO_3_ by the solid-phase method significantly improved the pigment properties of TiO_2_ in the same proportion.

## 1. Introduction

Perovskite (CaTiO_3_ or CaO·TiO_2_) and perovskite structural materials are an important class of oxides with an ABO_3_-type general formula. The Ca ion in CaTiO_3_ corresponds to the A cation in the general formula, which has a larger ionic radius and is surrounded by 12 oxygen anions to form a coordinated cubic octahedron, while the Ti ion that constitutes a coordinated octahedron with six oxygen anions occupies the B-site. These octahedrons spread into an extended three-dimensional network through corner-sharing. Perovskite has a density of 4.2 g/cm^3^, a hardness of 5.5, and a refractive index of 2.34 to 2.38 [1,2,3]. Generally, Ca and Ti in CaTiO_3_ are respectively replaced by other alkaline earth metal ions and transition metal ions to form perovskite structural materials. CaTiO_3_ and perovskite structural materials are stable in structure, and have good light absorption and photocatalytic properties, as well as excellent characteristics that can excite carriers and allow them to be separated and transported quickly [4,5,6]. Because of these features, perovskite materials are widely used in the fields of solar cells, photovoltaic devices, photodetectors and photocatalysis [7,8,9,10]. However, researchers have rarely studied perovskite as an inorganic pigment based on its higher optical refractive index or applied the pigment to products such as architectural coatings.

At present, the methods of synthesizing perovskite mainly include the solution cooling method, hydrothermal synthesis method, sol-gel method, and solid-phase sintering method. The solid-phase sintering method for synthesizing perovskite, which involves fully mixing raw materials (powder-like), high-temperature calcination and cooling, has obvious advantages, such as a simple and clean process [11,12,13,14]. Yet, since the relatively stable crystalline TiO_2_ (anatase or rutile) is used as the titanium source in this method, the reaction speed is slow and the cost is high [15]. Metatitanic acid is a precursor for the industrial production of TiO_2_ pigments by the sulfuric acid method, which is mainly composed of aqueous amorphous TiO_2_. It has the advantages of a large output, a low cost, a high purity and its reaction activity is higher than that of crystalline TiO_2_ [16,17,18]. Therefore, using metatitanic acid instead of the crystalline TiO_2_ as the raw material for the synthesis of perovskite will play a positive role in improving synthesis efficiency, saving energy and reducing costs.

For the above-mentioned background, synthetic perovskite powder (SPP) was herein synthesized by the solid-phase method using metatitanic acid (TiO_2_·nH_2_O) and calcium carbonate (CaCO_3_) as raw materials. We characterized the structure and morphology of SPP and studied its pigment properties and applications in architectural coatings.

## 2. Experiment Section 

### 2.1. Materials

The calcium carbonate in the experiment originated from ground calcium carbonate (GCC) produced by an enterprise in Enshi City, Hubei, China. X-ray diffraction (XRD) analysis showed that the GCC sample contained only one phase of calcite, indicating that the purity of the CaCO_3_ was high. After relevant characterization, we got the following data about the GCC sample: the whiteness was 96.0%, the oil absorption was 14.21/100 g, the hiding power was 165.0 g/m^2^, the medium diameter (D50) was 6.90 μm and the cumulative particle size distribution number of 90% (D90) was 27.16 μm. The experimental metatitanic acid (TiO_2_·nH_2_O) is the intermediate product of titanium dioxide produced by the industrial sulfuric acid method, which was provided by Henan Baililin Chemical Industry Co., Ltd. (Henan, China). Its chemical composition was 87.5% TiO_2_ and 12.5% H_2_O, of which TiO_2_ was mainly amorphous, and only a small amount was anatase. TiO_2_·nH_2_O had a whiteness of 92.40%, an oil absorption of 40.16/100g, a hiding power of 30.57 g/m^2^, and a median diameter (D50) of 0.76 μm. In addition, the main reagents used in this study were sodium polyacrylate, linseed oil and distilled water.

### 2.2. Synthesis of SPP and Preparing Architectural Coatings with SPP

The solid-phase synthesis of SPP was as follows: (1) GCC and TiO_2_·nH_2_O were weighed according to the molar ratio of CaO:TiO_2_ = 1:1, mixed and added with water to prepare a slurry with a solid content of 30%; (2) The slurry and grinding balls (diameter 1–3 mm, made of mullite) which accounted for three times the weight of the solids in the slurry were ground in a GSDM-3 1000 mL superfine stirring mill (Beijing Gosdel & Technology Co. Ltd., Beijing, China) for 60 min; (3) The ground CaCO_3_-TiO_2_·nH_2_O slurry was filtered, dried, calcined at a high temperature and scattered to obtain SPP. The calcination in the third step involved putting the ground CaCO_3_-TiO_2_·nH_2_O into a SX-4-10 box resistance furnace (Taisite Instruments Co., Ltd., Tianjin, China), raising the temperature to the required value at a heating rate of 5 °C/min, and then preserving it for a certain time. In order to explore the effect of the calcination temperature and time on the phases in SPP, we first calcined the ground CaCO_3_-TiO_2_·nH_2_O at different temperatures to obtain SPP-W, where W is the calcination temperature value. Then the ground CaCO_3_-TiO_2_·nH_2_O was calcined at 900 °C for different times to obtain SPP-900-T, where T is the calcination time value. For comparison, pure TiO_2_ pigments were prepared by directly calcining TiO_2_·nH_2_O at 900 °C.

The process of preparing architectural coatings with SPP as pigment was as described in [19]: (1) Raw materials were weighed according to the following coating formulation components and their proportions (mass fraction, wt%): 21 acrylic emulsion (AC-261); 21 water; 0.1 wetting agent; 0.64 dispersant (731A); 0.2 pH regulator; 0.24 defoamer (CF-754); 1.5 film-forming additive (C-12); 0.1 leveling agent (FSN); 0.72 leveling agent (2020); 2.2 thickener (DR-72); 4.8 talcum powder (23 μm); 7 kaolin (18 μm); 5 utron (a filler); 15 white pigment (SPP, pure TiO_2_ pigment, commercial titanium dioxide) and 20.5 GCC, total 100. (2) The components above were placed sequentially in a high-speed mixer to prepare the coating.

### 2.3. Structure Characterization and Performance of SPP

The phase components of SPP and other samples were tested and analyzed by XRD, using a D/MAX 2000/PC X-ray powder diffractometer (Rigaku Corporation, Tokyo, Japan) with Cu Kα radiation (λ = 0.15418 nm) generated at 35 kV and 50 mA. We used a speed of 2 °/min and a sampling interval of 0.02° to scan the sample between 10–80°. The structure and morphology of SPP were characterized by S-4800 scanning electron microscopy (SEM, Hitachi Electron Microscopy Company, Tokyo, Japan) with 10 kV acceleration voltage and 14.1 mm working distance and a Tecnai G2 F20 transmission electron microscope (TEM, FEI Company, The Netherlands) with 200 kV acceleration voltage.

The main pigment performance of SPP was evaluated by the indexes of whiteness, hiding power and oil absorption. Among them, the oil absorption was determined in accordance with the Chinese national standard GB/T 5211.15-2014 (General Methods of Test for Pigments and Extenders—Part 15: Determination of Oil Absorption). The hiding power was measured according to the Chinese national industry standard HG/T 3851-2006 (Covering Power Determination of Dyestuff) [20]. On the basis of the measured hiding power of SPP and pure TiO_2_ pigments (marked as C_SPP_ and C_T_ respectively), the relative hiding power of SPP was calculated, that is, the ratio of SPP’s hiding power to that of pure TiO_2_ (R, R = 100% × C_T_/C_SPP_). The part whose R value exceeded the TiO_2_ content in the SPP (58.8%) indicated an improvement in the hiding ability due to the conversion of TiO_2_ to perovskite (R, R = R − 58.8%).

The covering performance of architectural coatings prepared by adding SPP and other pigments was characterized by the coating film contrast ratio. A coating film with a thickness of 80 μm was prepared on a black and white plate using a BB-type wire rod applicator for coating materials. After being air-dried, the reflectance of the coating film in the black and white areas (recorded as F_b_ and F_w_) was measured with a C84-III reflectance tester (Shanghai Modern Environment Engineering Technique Co., Ltd., Shanghai, China) and converted into a contrast ratio (F_b_/F_w_).

## 3. Results and Discussion

### 3.1. Effect of Calcination Temperature and Time on the Phase of SPP Particles

**Figure 1:**
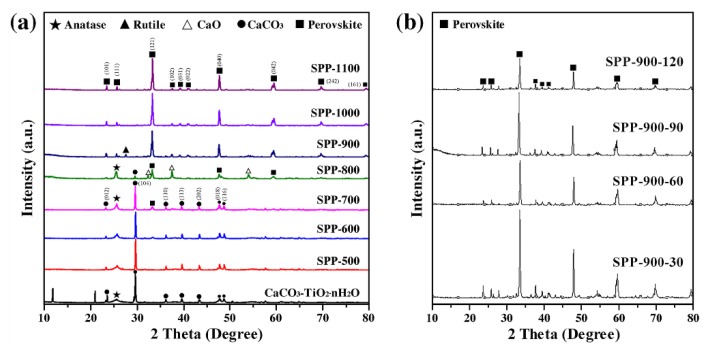
XRD patterns of (**a**) CaCO_3_-TiO_2_·nH_2_O calcined at different temperatures and (**b**) for different times (SPP-900).

The XRD patterns of the ground CaCO_3_-TiO_2_·nH_2_O and its products calcined at different temperatures (SPP-W) for 30 min are shown in Figure 1a. In the ground CaCO_3_-TiO_2_·nH_2_O, the diffraction peaks of calcite and anatase appear, reflecting the characteristics of anatase in TiO_2_·nH_2_O and CaCO_3_ (JCPDS No. 05-0586) in the GCC. The phase compositions of SPP-500 and SPP-600 are the same as that of the ground CaCO_3_-TiO_2_·nH_2_O, which indicated that calcination at a lower temperature cannot generate a new phase. Increasing the temperature to 700 °C, in addition to calcite and anatase, causes a weak diffraction peak of perovskite to appear in the XRD pattern. This may be due to the reaction of calcite and anatase synthesizing perovskite at the interface between them. Meanwhile, the SPP-800 particles were composed of perovskite, anatase and CaO, and the diffraction peak of calcite in the pattern disappeared, showing that the decomposition reaction of CaCO_3_ occurred (the decomposition products were CaO and CO_2_). As the temperature further increased to 900–1100 °C, both the anatase and CaO in the XRD patterns of the calcined products disappeared. The phases of SPP-900, 1000 and 1100 were completely composed of perovskite (JCPDS No. 22-0153) and the diffraction peaks were significantly enhanced compared with the calcined products below 900 °C, which demonstrated that we synthesized the SPP with a single perovskite phase at 900–1100 °C.

The XRD patterns of the ground CaCO_3_-TiO_2_·nH_2_O calcined at 900 °C for different times (SPP-900-T) are displayed in Figure 1b. Although the heating time was increased from 30 to 120 min, the obtained products were all single phases of perovskite, and increasing the calcination time had no effect on the phase composition of SPP-900. Considering this comprehensively, we selected a calcination temperature of 900 °C and a time of 30 min as the optimal conditions for the synthesis of SPP.

**Figure 2:**
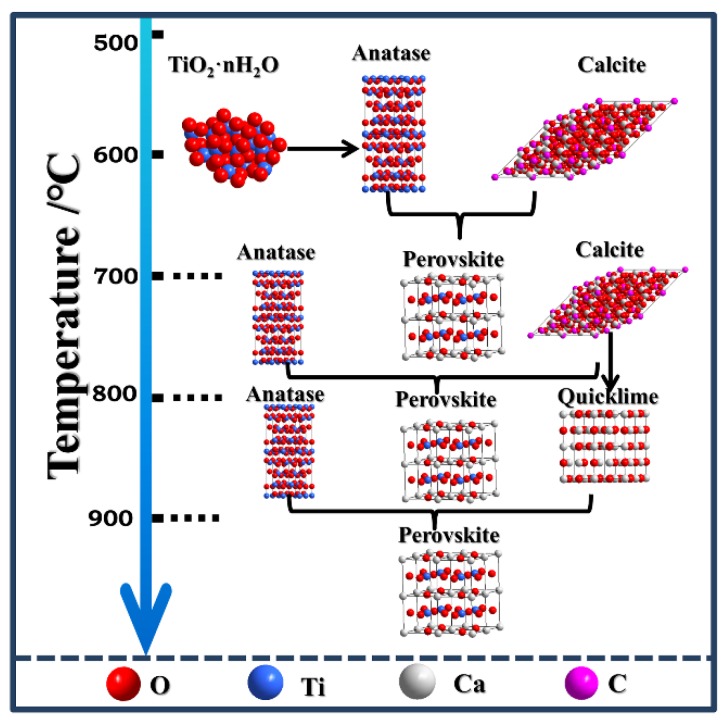
Schematic diagram of perovskite phase transformations during the synthesis of SPP.

The transformation of CaCO_3_-TiO_2_·nH_2_O phases, along with the calcination temperature and the process of finally synthesizing the perovskite, are exhibited in Figure 2. This diagram shows that when the calcination temperature was raised to 700 °C, a small amount of perovskite was generated due to the reaction between CaCO_3_ and TiO_2_. When the temperature continued to increase, CaCO_3_ decomposed to form CaO, and the final perovskite was formed by the reaction of CaO and TiO_2_. The reaction equations at different temperatures are provided as follows:

$$\begin{array}{c} 700{}^{\circ}C:TiO_{2}+CaCO_{3}\to CaTiO_{3}+CO_{2}↑ \end{array}$$

$$\begin{array}{c} 800{}^{\circ}C:CaCO_{3}\to CaO+CO_{2}↑ \end{array}$$

$$\begin{array}{c} 900{}^{\circ}C:TiO_{2}+CaO\to CaTiO_{3} \end{array}$$

**Figure 3:**
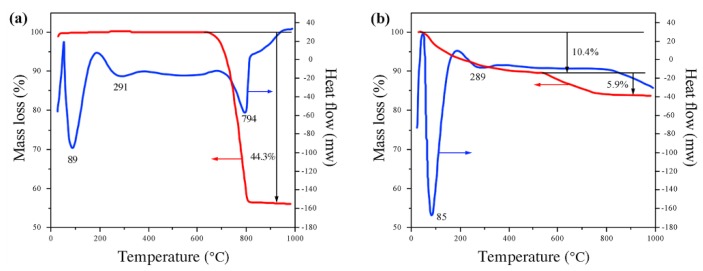
TG/DSC curves of (**a**) CaCO_3_ and (**b**) TiO_2_·nH_2_O.

Figure 3 shows the TG/DSC curves of CaCO_3_ and TiO_2_·nH_2_O. In Figure 3a, the endothermic peak and 44.3% weight loss of CaCO_3_ appeared at 794 °C, corresponding to the phenomenon of CO_2_ release due to its decomposition. It can be observed from Figure 3b that TiO_2_·nH_2_O began its endothermic peak from 289 °C and gradually lost weight between 550–800 °C, which is attributed to the loss of structured water in TiO_2_·nH_2_O. The exothermic peak at about 800 °C indicates a phase transition of TiO_2_ from anatase to rutile. These features are consistent with the XRD results in Figure 1.

### 3.2. Effect of Calcination Temperature on the Morphology of SPP Particles

**Figure 4:**
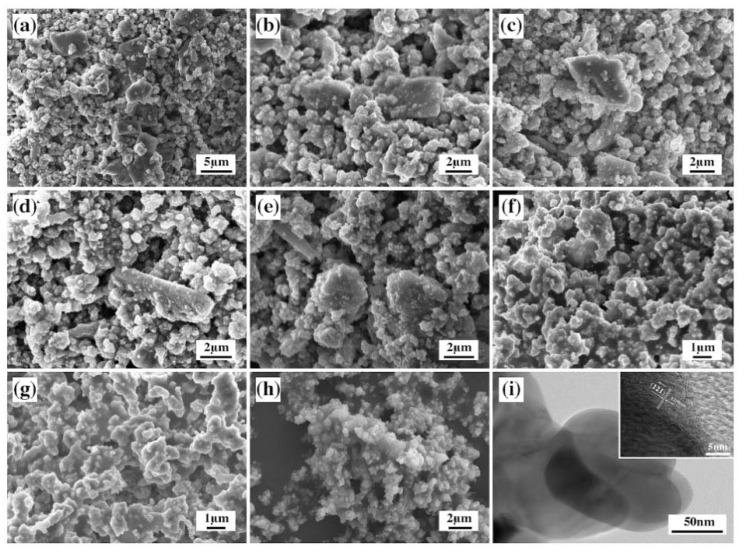
SEM images of (**a**) CaCO_3_-TiO_2_·nH_2_O and (**b**–**h**) its calcined products at different temperatures. (**b**) 500 °C; (**c**) 600 °C; (**d**) 700 °C; (**e**) 800 °C; (**f**) 900 °C; (**g**) 1000 °C; (**h**) 1100 °C. (**i**) TEM and HRTEM (inset) images of SPP-900.

Figure 4 shows SEM images of the ground CaCO_3_-TiO_2_·nH_2_O calcined at different temperatures for 30 min. In Figure 4a, the CaCO_3_-TiO_2_·nH_2_O without calcination consisted of two kinds of particles with different morphologies and sizes. The larger particles with a particle size of 3–6 μm and an irregular block shape were CaCO_3_, while the smaller particles with a size of 0.5–1 m and a uniform distribution were TiO_2_·nH_2_O, exhibiting the composition characteristics of GCC and TiO_2_·nH_2_O in the raw materials. The morphology of CaCO_3_-TiO_2_·nH_2_O particles calcined at 500, 600, and 700 °C in Figure 4b–d is basically the same as the raw materials that were not calcined in Figure 4a, revealing that there was no formation of a new phase at a low calcination temperature. Unlike the above samples, although the size of the large particles in SPP-800 remained unchanged, their edges and corners mostly disappeared, and the appearance became mellowed (Figure 4e). This phenomenon was attributed to the thermal decomposition of CaCO_3_ in the raw materials to generate CaO at this temperature. It can be seen from Figure 4f–h that the particle morphology of SPP-900, SPP-1000 and SPP-1100 was granular and long columnar, with the size of about 1–2 μm. In these three samples, the SPP-900 particles had better dispersibility, while the SPP-1000 and SPP-1100 particles agglomerated with each other (the latter had a stronger aggregation behavior.) This is the manifestation of CaCO_3_-TiO_2_·nH_2_O calcined at 900–1100 °C to form perovskite. The conclusions obtained from the SEM image analysis in this section were consistent with the above XRD results.

Figure 4i is the TEM image of SPP-900 (CaCO_3_-TiO_2_·nH_2_O calcined at 900 °C) synthesized by the solid-phase method. It can clearly be observed that the perovskite unit particles were ellipsoidal, and their size was about 50–150 nm. The Figure 4i inset is the HRTEM image of SPP-900, displaying its clear lattice fringes. We measured an interval scale of 0.2701 nm, which corresponded to the interplanar spacing of the (121) plane of the perovskite crystals, further confirming the result of XRD that SPP was composed of a perovskite phase [21].

### 3.3. Pigment Properties of SPP Particles

**Figure 5:**
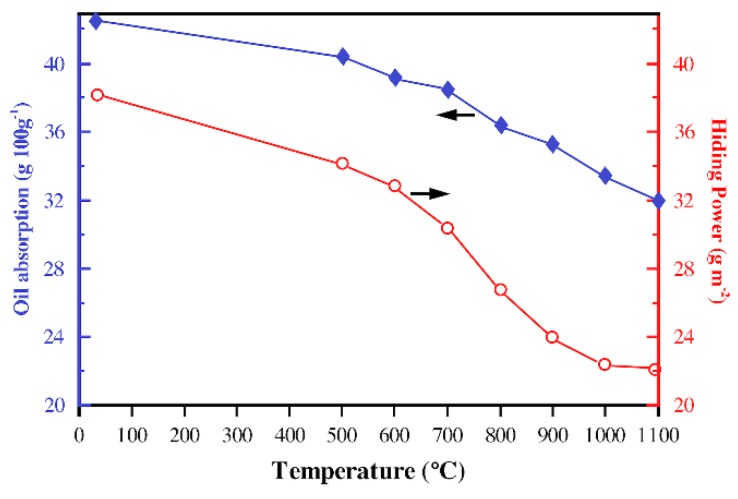
Oil absorption (rhombus) and hiding power (hollow circle) of CaCO_3_-TiO_2_·nH_2_O calcined at different temperatures.

Figure 5 shows the hiding power and oil absorption of CaCO_3_-TiO_2_·nH_2_O calcined at different temperatures. The hiding power of SPP particles decreased from 38 to 24 g/m^2^ by degrees with the increase in calcination temperature (from 25 to 900 °C), which means that its hiding performance gradually increased. When the temperature was higher than 700 °C, the magnitude of the enhancement became significantly larger, which was apparently caused by the presence of perovskite in the calcined products and its growing content. When the temperature was raised to 1000 and 1100 °C, we discovered that the hiding power of the SPP drops to about 22.5 g/m^2^, that is, the hiding performance continues to increase and reaches a stable value, which should be the result of the SPP having only a perovskite phase and an increased degree of crystallization. Figure 5 also describes the tendency of the SPP’s oil absorption to gradually decrease with the increase in the calcination temperature, where the oil absorption of the SPP at a temperature higher than 900 °C was less than 35 g/100 g.

The comparison of the pigment performance between SPP-900, pure TiO_2_ pigment (TiO_2_·nH_2_O calcined at 900 °C) and raw GCC is shown in Table 1. Clearly, the hiding power values of the raw GCC and pure TiO_2_ pigments are 165.00 g/m^2^ and 19.60 g/m^2^, respectively, indicating that the GCC had no hiding ability while the pure TiO_2_ pigment had a strong hiding ability. The hiding power of the SPP synthesized by the solid-phase method was 24.02 g/m^2^, which was close to that of the pure TiO_2_ pigment and much stronger than the experimental data of the raw GCC. An in-depth analysis of these data shows that the hiding power of SPP reached 81.6% (R, relative hiding power) of the pure TiO_2_ pigment, which was 22.8% higher than the proportion of TiO_2_ contained in perovskite (58.8%). The results show that the synthesis of perovskite by using CaCO_3_ and TiO_2_·nH_2_O as raw materials significantly improved the hiding performance of TiO_2_, which was the reason why SPP had the same hiding performance as the pure TiO_2_ pigment. Besides, the oil absorption of SPP was close to that of TiO_2_ pigments (values were 35.03 and 33.26 g/100 g, respectively). Therefore, SPP is considered to have comprehensive properties comparable to pure TiO_2_ pigments.

### 3.4. Coating Performance with SPP as a Pigment

Based on the formula and steps in Section 2.2, we obtained the contrast ratio of architectural coating films prepared with SPP, pure TiO_2_ pigment and commercial titanium dioxide (rutile) as white pigments. The coating film contrast ratios of these three coatings all exceeded 0.90, meeting the requirements of the Chinese National Standard GB/T 9755-2014 [22]. The contrast ratio of SPP’s coating film was 0.92, which was slightly lower than that of rutile titanium dioxide coating (0.93), but larger than the value of pure TiO_2_ pigment (0.91), demonstrating that the SPP had reached the level of titanium dioxide with the same proportion, so it can be used as a pigment for architectural coatings. The contrast ratio is an index reflecting the opacity of the coating film, which mainly depends on the properties of the pigment in it, including oil absorption and hiding power. The contrast ratio of the coating with SPP was equivalent to the coating with pure TiO_2_ pigment or rutile titanium dioxide, and this confirmed that SPP had the same performance as pure TiO_2_ pigment and rutile titanium dioxide.

## 4. Conclusions

Using metatitanic acid (TiO_2_·nH_2_O) and calcium carbonate (CaCO_3_) as raw materials, synthetic perovskite powder (SPP) with a single perovskite phase was synthesized by the solid-phase method at a calcination temperature of 900–1100 °C. The process of generating perovskite with an increasing calcination temperature involved a small amount of perovskite initially formed by the reaction of CaCO_3_ and TiO_2_, and after the decomposition of CaCO_3_ into CaO and CO_2_, the subsequent perovskite was formed by the reaction between CaO and TiO_2_. Particles of SPP, with a unit particle size of 50–150 nm and an aggregate size of 1–2 μm, were well dispersed. The whiteness of the SPP was 90.5%.

The synthesized SPP had a pigment performance equivalent to that of pure TiO_2_. Its oil absorption was 35.03 g/100 g. Its hiding power was 24.02 g/m^2^ and this value reached 81.6% of TiO_2_ pigment, which is 22.8% higher than the proportion of TiO_2_ in perovskite. Adding SPP to architectural coatings has a coating film contrast ratio of 0.92, which matches the use of rutile titanium dioxide, and meets the Chinese National Standard (GB/T 9755-2014).

## Figures and Tables

**Table 1:** Pigment performance comparison of SPP, pure TiO_2_ pigments and raw GCC.

Samples	Oil Absorption/g·100 g^−1^	Whiteness/%	Hiding Power/g·m^−2^	Relative Hiding Power (R)/%	(R = R − 58.8)/%
SPP	35.03	90.5	24.02	81.60	22.80
Pure TiO_2_	33.26	87.8	19.60	100	--
Raw GCC	14.21	96.2	165.00	11.88	--
